# Shorter anogenital distance is observed in patients with testicular microlithiasis using magnetic resonance imaging

**DOI:** 10.1186/s13244-021-00989-5

**Published:** 2021-04-12

**Authors:** Malene Roland Vils Pedersen, Palle Jørn Osther, Søren Rafael Rafaelsen

**Affiliations:** 1 Department of Radiology, Vejle Hospital, University of Southern Denmark, Beriderbakken 4, Vejle, Denmark; 2 Institute of Regional Health Research, University of Southern Denmark, Campusvej 55, Odense, Denmark; 3 Urological Research Center, Department of Urology, Vejle Hospital, University of Southern Denmark, Beriderbakken 4, Vejle, Denmark

**Keywords:** Testicular microlithiasis, Magnetic resonance imaging, Anogenital distance, Andrology

## Abstract

**Objective:**

To investigate the anogenital distance in patients with and without testicular microlithiasis (TML).

**Methods:**

A total of 101 patients underwent a conventional standard clinical B-mode scrotal ultrasonography and scrotal MRI. The patients were divided into two groups: patients with TML and non-TML. The latter served as control group. The anogenital distance was measured by a straight line from center of the anus to the posterior base of scrotum using MRI.

**Results:**

In the TML group, mean AGD was 5.4 (± 1.07) cm (range 29–79 mm), and in non-TML 5.9 (± 1.03) cm (range 35–85 mm) (*p* = 0.04).

**Conclusion:**

MRI is a useful tool to measure the AGD. It is easy to perform without any discomfort to patients. We found AGD to be lower in patients with TML.

## Key points

1. MRI has been proposed as a technique to measure the anogenital distance.
2. Testicular Microlithiasis has shorter anogenital distance compared to men without microlithiasis.
3. Anogenital distance measurements allows the measurement to be performed without any discomfort to the patient.

## Introduction

Testicular microlithiasis (TML) is characterized sonographically by the presence of very small echogenic foci diffusely dispersed throughout the testicular parenchyma without acoustic shadowing. Microliths are typically 1 up to 3 mm in size and may appear both unilaterally and bilaterally. TML is of unknown origin and is an asymptomatic condition. It is unknown how and why TML develops, but the condition can be present in all age groups.

TML is diagnosed by ultrasound and is not visible by other image modalities such as Computed Tomography or Magnetic Resonance Imaging (MRI). In 1987, TML was described as “innumerable tiny bright echoes diffusely and uniformly scattered” [1] throughout the testicular tissue. TML has been associated with different testicular pathologies, e.g., Klinefelter syndrome, cryptorchidism (undescended testis), infertility, ethnicity, testicular dysgenesis syndrome (TDS) and increased risk of intratubular germ cell neoplasia [2–9].

The anogenital distance (AGD) is defined as the distance between the anus to the genitals. It has been suggested that shorter AGD is associated with infertility and testicular function [10–12] and infertility by prenatal exposure to smoking [13]. Studies have reported children with cryptorchidism have shorter AGD compared to healthy controls [14, 15]. A recent study found paracetamol exposure between 8 and 14 weeks of gestation was significant associated with shorter AGD [16]. AGD has also been suggested to be part of the TDS linked to abnormal testicular development during fetal life [17]. To our knowledge, no previous studies have investigated the association between TML and the AGD. Thus, based on these considerations, TML and AGD may be interrelated, and the aim of this retrospective explorative study was to compare AGD in patients with and without TML.

## Methods

The study was approved by the local hospital review board and the Danish Data protection Agency. Data were gathered according to the principles in the Declaration of Helsinki. Informed consent prior examination was mandatory.

### Testicular microlithiasis and non-testicular microlithiasis patients

All patients were referred by their general practitioner to the department of Radiology due to testicular discomfort, pain, swelling or a small palpable lump and received a conventional standard B-mode scrotum ultrasonography and scrotal MRI examination during the period 2013–2017.

The subjects were divided into two groups: TML group and non-TML group. The latter served as control group. TML was defined as hyperechogenic foci with no acoustic shadowing, between 1 and 3 mm in size, and with five or more foci per field of view. The European Society of Urogenital Radiology (ESUR) recommends the definition of five or more foci per field of view [18].

Non-TML patients were defined as patients with normal testicles tissue without TML.

### Ultrasonography

All patients underwent a standard scrotal ultrasound investigation at our department of Radiology. The scrotal ultrasonography was performed by two of the authors or four other certified radiologists (with more than five years of scrotal ultrasound experience). Data were stored in the department's PACS system (Picture Archive Communication System, Easyviz Impax workstation, Medical Insight, Valby, Denmark). The patients were placed supine with the penis placed against the abdominal wall. First, palpation of both testicles was performed. Ultrasonography was used to measure length, height and width of both testicles. The presence or absence of TML was noted. The patients were investigated with a Siemens S3000 machine (Acuson Corporation, Siemens, Mountain View, CA, USA) with a linear 9L4 probe, frequency bandwidth: 4–9 MHz.

### MRI and AGD measurements

**Fig. 1:**
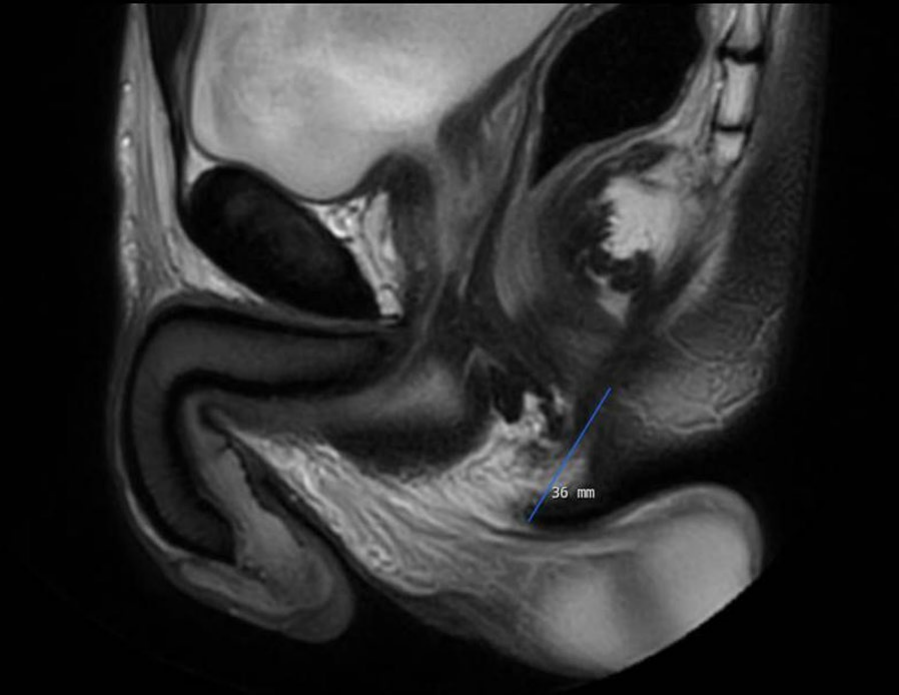
shows a sagittal MRI image of non-TML patient with AGD measurement of 36 mm. The measurement was performed using a straight line from the center of the anus to the posterior base of scrotum

All patients underwent MRI scrotal examination by a 1.5-T unit (Phillips, Ingenia, release 4.1.3.3) using a posterior coil. The patients were placed prone during the MRI scan, in order to limit scrotum movement. First, a short overview survey was performed followed by a T2-weighted spin echo; a T1-weighted and diffusion weighted scans. The MRI scan time was maximum 20 min, and the MRI protocol was developed for imaging the scrotum (Table 1). AGD is defined as center of anus to the posterior base of scrotum using a straight line (Fig. 1).

**Table 1:** The MRI examination protocol

Parameters	T2	T1	DWI
TR/TE	2593/100	650/10	3224/108
WFS/BW	0.932/235.2	0.815/266.5	13.121/16.6
FOV (mm)	130 × 130	110 × 110	200 × 200
Slice thickness	2 mm	2 mm	3 mm
Matrix	164 × 162	140 × 137	124 × 122
Acq voxel	0.79 × 0.45 × 2	0.79 × 0.80 × 2	1.61 × 1.63 × 3
Recon voxel	0.45 × 0.45 × 2	0.69 × 0.69 × 2	1.56 × 1.56 × 3
NSA	3	3	4
Scan time	4.14 min	5.00 min	6.04 min
Slice gab	0.9 mm	0.9 mm	0.4 mm
b- values	NR	NR	0, 100, 300, 600, 900, 1100
Scan plane	Axial	Axial	Axial

DWI = diffusion-weighted imaging, TE = Time to echo; TR = time to repetition; WFS = water fat shift, BW = bandwidth; FOV = field of view; NSA = numbers of averages, NR = not relevant. Acq = acquisition

One observer measured the AGD distance twice in all patients in order to obtain valid and consistent measurements including intra-observer variability. The observer was blinded to patient data, patient history and other examinations. The same diagnostic screen was used to perform all the measurements. The observer could not discuss the cases with colleagues, as the measurements were performed in an undisturbed room. The observer had a time interval of 4 months between the first and second AGD measurements.

### Statistics

All analyses were performed with STATA statistical software (version 15.1 STATA Corporation, College station TX, USA). Values of *p* ≤ 0.05 were considered significant.

Association between TML and non-TML was assessed using Chi-square test for categorical variables and Mann–Whitney U-test for continuous variables. Continuous data were summarized using mean, and categorical data summarized by frequency distributions. Normal distribution of AGD measurements was tested with Shapiro–Wilk test. AGD measurements were not normally distributed.

Interclass correlation (ICC) estimate, and 95% confidence interval based on two-way random-effects model.

## Results

Figure 1 demonstrates how the AGD measurements were performed using a straight line from the center of the anus to the posterior base of the scrotum using a sagittal MRI image.

**Fig. 2:**
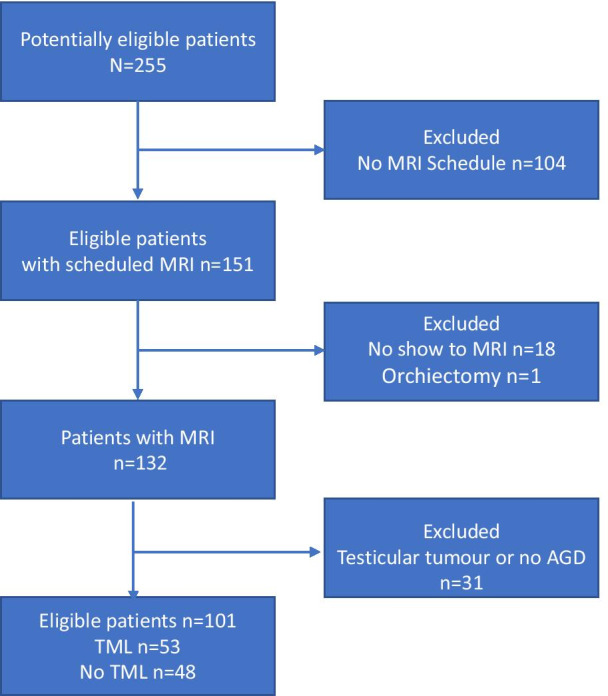
Patient flow diagram

One hundred and one male patients were included (patient flow diagram, Fig. 2). The patients were divided into a TML and non-TML group. The TML-group included 53 (52%) patients and the non-TML included 48 (48%) patients. Nineteen (35.8%) TML patients were diagnosed with bilateral TML.

The mean age was 46.8 years in all patients (range 23–80 years). The mean age in the TML-group was 46.9 (± 13.1, range 23–70 years), and mean age in the non-TML-group was 46.6 years (± 14.0; range 23–80 years). There was no statistical difference in age groups between the two groups (*p* = 0.71).

In the TML-group, the AGD mean was 5.4 (± 1.07) cm (range 29–79 mm), and in the non-TML group the AGD was 5.9 (± 1.03) cm (range 35–85 mm). Table 2 shows association between TML and non-TML subjects. The AGD was lower in TML patients compared to Non-TML patients (*p* = 0.04). There were no differences between the two groups on the presence of testicular cyst, hernia, varicocele, spermatocele or hydrocele.

**Table 2:** Association between TML and non-TML subjects

Variable	TML *n* = 53 (%)	Non-TML *n* = 48 (%)	*p* value*
Age			0.71
< 30	5 (9.4)	4 (8.3)	
30–39	12 (22.6)	14 (29.1)	
40–49	14 (26.4)	15 (31.2)	
50 +	22 (41.6)	15 (31.2)	
Varicocele			0.91
Unilateral	7 (13.2)	5 (10.4)	
Bilateral	3 (5.7)	3 (6.3)	
None	43 (81.1)	40 (83.3)	
Hydrocele			0.22
Unilateral	16 (30.2)	8 (16.7)	
Bilateral	10 (18.9)	8 (16.7)	
None	27 (50.9)	32 (66.6)	
Spermatocele			0.74
Unilateral	13 (24.5)	11 (22.9)	
Bilateral	4 (7.6)	2 (4.2)	
None	36 (67.9)	35 (72.9)	
Hernia			0.07
Yes	0 (0)	3 (6.3)	
No	53 (100)	45 (93.7)	
Cyst			0.19
Yes	2 (3.8)	5 (10.4)	
No	51 (96.2)	43 (89.6)	

^*^Chi-squared test

The intra-observer variability measuring the AGD was very good with an ICC of 0.92 (5% confidence interval 0.883–0.947).

## Discussion

In this study, AGD was lower in the TML group compared to non-TML group (*p* = 0.04). TDS suggests abnormal development of the male reproductive system during fetal life could be a disorder that becomes clinically apparent during infancy or adulthood [17]. TDS includes cryptorchidism, poor semen quality, low testicular volume, infertility and testicular cancer.

The association between TML and infertility is not well understood. The pathogenesis of TML is considered to be intratubular obstructions of the seminiferous tubules. Yee et al. suggested that decreased fertility may be expected in patients with TML as 30–60% of the seminiferous tubules may be obstructed by intratubular concretions [19]. This study does not investigate male infertility. Still, AGD is of great importance for the public health as AGD has potential to become a biomarker. Especially as AGD appears to be constant during adulthood [20].

No difference was found between TML and non-TML and common testicular conditions, e.g., varicocele, spermatocele and hydrocele. This suggests that TML may not affect the common testicular conditions. However, the difference in AGD 5.4 cm versus 5.9 could be caused by chance or by a small sample size. Therefore, more studies are warranted to confirm this finding.

Intra-observer measurement error variability was excellent, showing that AGD obtained by MRI can be assessed reliable. Studies focusing on AGD in adults are limited, since most studies have investigated infants and young children. Furthermore, there is no gold standard concerning measurement of AGD in adult males. Yet, two studies investigating interobserver agreement in adult males have found good and very good interclass correlation of 0.80 and 0.932, respectively [21, 22]. Both studies used a stainless-steel digital calliper. This demonstrates that AGD is an easily obtained measurement but using calliper is not a patient friendly investigation. We also found an excellent interclass correlation (ICC 0.92). Measurement using MRI has the advantage that there is no risk of movement during the alignment; furthermore, it is an easy, and repeatable tool. In general, MRI of the scrotum is seldomly performed in clinical practice, mostly due to scrotal ultrasonography is an easier and cheaper modality. However, as demonstrated in the present study, MRI provides high-quality image, and AGD is easily measured. Additionally, measuring AGD by MRI is more accepted by the patients, as the measurement is done electronically and retrospective. We advocate that MRI is a feasible tool to measure AGD, and it may be considered as a clinical tool when investigating infertility in the future.

## Limitation

Potential limitations in this study may be that the cohort was based on symptomatic patients', and therefore, the included patients may not represent the general population. However, many men experience scrotal symptoms during their lifetime. Another limitation was prone position during MRI. This may affect the AGD measurements, as the scrotum may be more compact, but one could argue that the AGD will be more precise as the prone position limits movement of the scrotum. Nevertheless, this study is the first to suggest MRI as a tool to measure the AGD length. This method has some limitation and cannot be transferred directly into clinical practice because scrotal MRI is not a widely used examination available. Furthermore, compared to ultrasound it is an expensive examination. This will potentially limit the use in daily clinical practice. However, this study showed that AGD is easily obtained.

## Conclusion

This study provides new knowledge about AGD in men with TML. MRI provides AGD measurement without any discomfort to patients and is easy to perform. AGD can be MRI monitored in different groups in the future. Potentially, men under elucidation for infertility may benefit from this modality. The AGD was lower in TML patients compared to Non-TML patients (*p* = 0.04); however, more studies are warranted.

## Data Availability

All data generated or analyzed during this study are included in this manuscript.

## Abbreviations

AGD: The anogenital distance
ICC: Interclass correlation
MRI: Magnetic Resonance Imaging
PACS: Picture Archive Communication System
TDS: Testicular dysgenesis syndrome
TML: Testicular microlithiasis

## References

[1] Doherty FJ, Mullins TL, Sant GR, Drinkwater MA, Ucci AAJ (1987) Testicular microlithiasis: a unique sonographic appearance. J Ultrasound Med 6:389–392 10.7863/jum.1987.6.7.389 3302309

[2] Fedder J, Gravholt CH, Kristensen SG (2015). Testicular sperm sampling by subcapsular orchiectomy in Klinefelter patients: a new simplified treatment approach. Urology.

[3] Goede J, Hack WWM, van der Voort-Doedens LM, Pierik FH, Looijenga LHJ, Sijstermans K (2010). Testicular microlithiasis in boys and young men with congenital or acquired undescended (ascending) testis. J Urol.

[4] Aizenstein RI, DiDomenico D, Wilbur AC, O´Neil HK (1998) Testicular microlithiasis: association with male infertility. J Clin Ultrasound 26:195–198 10.1002/(sici)1097-0096(199805)26:4<195::aid-jcu3>3.0.co;2-8 9572382

[5] Pedersen MR, Bartlett EC, Rafaelsen SR (2017). Testicular microlithiasis is associated with ethnicity and socioeconomic status. Acta Radiol Open.

[6] van Casteren NJ, Looijenga LH, Dohle GR (2009). Testicular microlithiasis and carcinoma in situ overview and proposed clinical guideline. Int J Androl.

[7] Peterson AC, Bauman JM, Light DE, McMann LP, Costabile RA (2001). The prevalence of testicular microlithiasis in an asymptomatic population of men 18 to 35 years old. J Urol.

[8] Dantsev IS, Ivkin EV, Tryakin AA (2018). Genes associated with testicular germ cell tumors and testicular dysgenesis in patients with testicular microlithiasis. Asian J Androl.

[9] Skakkebæk NE, Rajpert-De Meyts E, Main KM (2001). Testicular dysgenesis syndrome: an increasingly common developmental disorder with environmental aspects. Hum Reprod.

[10] Eisenberg ML, Lipshultz LI (2015). Anogenital distance as a measure of human male fertility. J Assist Reprod Genet.

[11] Mendiola J, Stahlhut RW, Jorgensen N, Liu F, Swan SH (2011). Shorter anogenital distance predicts poorer semen quality in young men in Rochester. Environ Heal Perspect.

[12] Eisenberg ML, Jensen TK, Walters RC, Skakkebaek NE, Lipshultz LI (2012). The relationship between anogenital distance and reproductive hormone levels in adult men. J Urol.

[13] Virtanen HE, Sadov S, Toppari J (2012). Prenatal exposure to smoking and male reproductive health. Curr Opin Endocrinol Diabetes Obes.

[14] Thankamony A, Lek N, Carroll D (2013). Anogenital distance and penile length in infants with hypospadias or cryptorchidism: comparison with normative data. Environ Health Perspect.

[15] Hsieh MH, Breyer BN, Eisenberg ML, Baskin LS (2008). Associations among hypospadias, cryptorchidism, anogenital distance, and endocrine disruption. Curr Urol Rep.

[16] Fisher BG, Thankamony A, Huges IA, Ong KK, Dunger DB, Acerini CL (2016). Prenatal paracetamol exposure is associated with shorter anogenital distance in male infants. Hum Reprod.

[17] Skakkebaek N, Rajpert-De Meyts E, Main K (2001). Testicular dysgenesis syndrome: an increasingly common developmental disorder with environmental aspects. Hum Reprod.

[18] Richenberg J, Belfield J, Ramchandani P (2015). Testicular microlithiasis imaging and follow-up: guidelines of the ESUR scrotal imaging subcommittee. Eur Radiol.

[19] Yee WS, Kim YS, Kim SJ, Choi JB, Kim SI, Ahn HS (2011). Testicular microlithiasis: prevalence and clinical significance in a population referred for scrotal ultrasonography. Korean J Urol.

[20] Eisenberg M, Hsieh T-C, Lipshultz LI (2013). The relationship between anogenital distance and age. Andrology.

[21] Mendiola J, Onate-Celdrán J, Samper-Mateo P (2016). Comparability and reproducibility of adult male anogenital distance measurements for two different methods. Andrology.

[22] Foresta C, Valente U, Di Nisio A (2018). Anogenital distance is associated with genital measures and seminal parameters but not anthropometrics in a larger cohort of young adult men. Hum Reprod.

